# Reproducibility of wrist home blood pressure measurement with position sensor and automatic data storage

**DOI:** 10.1186/1471-2261-9-20

**Published:** 2009-05-27

**Authors:** Sakir Uen, Rolf Fimmers, Miriam Brieger, Georg Nickenig, Thomas Mengden

**Affiliations:** 1 Department of Cardiology and Vascular Medicine, University of Bonn, Bonn, Germany; 2 Department of Biometry and Medical Statistics, University of Bonn, Bonn, Germany

## Abstract

**Background:**

Wrist blood pressure (BP) devices have physiological limits with regards to accuracy, therefore they were not preferred for home BP monitoring. However some wrist devices have been successfully validated using etablished validation protocols. Therefore this study assessed the reproducibility of wrist home BP measurement with position sensor and automatic data storage.

**Methods:**

To compare the reproducibility of three different(BP) measurement methods: 1) office BP, 2) home BP (Omron wrist device HEM- 637 IT with position sensor), 3) 24-hour ambulatory BP(24-h ABPM) (ABPM-04, Meditech, Hun)conventional sphygmomanometric office BP was measured on study days 1 and 7, 24-h ABPM on study days 7 and 14 and home BP between study days 1 and 7 and between study days 8 and 14 in 69 hypertensive and 28 normotensive subjects. The correlation coeffcient of each BP measurement method with echocardiographic left ventricular mass index was analyzed. The schedule of home readings was performed according to recently published European Society of Hypertension (ESH)- guidelines.

**Results:**

The reproducibility of home BP measurement analyzed by the standard deviation as well as the squared differeces of mean individual differences between the respective BP measurements was significantly higher than the reproducibility of office BP (p < 0.001 for systolic and diastolic BP) and the reproducibility of 24-h ABPM (p < 0.001 systolic BP, p = 0.127 diastolic BP). The reproducibility of systolic and diastolic office versus 24-h ABPM was not significantly different (p = 0.80 systolic BP, p = 0.1 diastolic BP). The correlation coefficient of 24-h ABMP (r = 0.52) with left ventricular mass index was significantly higher than with office BP (r = 0.31). The difference between 24-h ABPM and home BP (r = 0.46) was not significant.

**Conclusion:**

The short-term reproducibility of home BP measurement with the Omron HEM-637 IT wrist device was superior to the reproducibility of office BP and 24- h ABPM measurement. Furthermore, home BP with the wrist device showed similar correlations to targed organ damage as recently reported for upper arm devices. Although wrist devices have to be used cautious and with defined limitations, the use of validated devices with position sensor according to recently recommended measurement schedules might have the potential to be used for therapy monitoring.

## Background

The reproducibility of office blood pressure (office BP) measurement in clinical studies as well as in doctor's offices is very limited for various reasons. These include for example observer bias, the white-coat and placebo effects [1]. The poor reproducibility of office BP means that a much larger number of patients must be included in clinical pharmacological studies owing to the high individual variability between two office BP measurements. This is a prerequisite for demonstrating for example the therapeutic effects of antihypertensive agents. Incontestably, the ambulatory 24-hour blood pressure measurement (24-h ABPM) shows much better reproducibility than the office BP measurements [2]. As a result of the larger number of measurements and the better standardization, the 24-h ABPM reflects end organ damage better than office BP, for example left ventricular hypertrophy [3]. Multiple self-measurements carried out by the patients at their home (home BP) improve the reproducibility of blood pressure measurements substantially compared to office BP [4]. The development of more precise measurement technologies, clinical validation protocols as well as increasing standardization of the measurement technique and the measurement protocol can raise the reproducibility of home BP measurements even further [5]. BP measurement in the wrist provides several advantages especially when performed by the patient at home. For example, it does not require taking off clothes for cuff application, the cuff can be applied more easily, and the readings are less sensitive to obesity because wrist size is little affected by obesity [6]. On the other hand accuracy of wrist devices may be limited by several factors such as anatomy of the wrist and arteriosclerotic vascular changes as pointed out in a recent paper by Kikuya et al [7].

It has so far been unclear whether the reproducibility of home BP measurement on the wrist is comparable with that of 24-hour on the upper arm. Moreover, there have been as yet not been any studies investigating the correlation of wrist measurements with end organ damage such as left ventricular hypertrophy.

In the present study, the following questions were therefore investigated with the Omron wrist instrument HEM-637 IT with a position sensor:

1. To what extent is the short-term reproducibility of wrist measurements with a position sensor comparable with that of 24-h ABPM and office BP measurement?

2. How do multiple wrist measurements correlate with left ventricular mass index (LVMI) as compared to 24-h ABPM and office BP measurement?

## Methods

The objective of the study was to investigate the reproducibility of office BP, home BP and 24-h ABPM over a period of one week. For this purpose, an office BP measurement was taken on study days 1 and 7 in 101 patients and an ambulatory 24-h ABPM was carried out on study days 7 and 14. In the time intervals between study days 1 and 7 and between study days 8 and 14, the patients measured their home BP themselves with an oscillometric wrist instrument.

### Study population

One hundred and one participants were included in the study. All subjects were consecutively recruited from the outpatients of Medical Policlinic of the University of Bonn.

The data of 97 patients with complete data sets (55 men and 42 women, 69 participants with office-measured hypertension, 28 with office-measured normotension, 23 participants with antihypertensive treatment and 74 without treatment) were included in the statistical analysis. Hypertensive subjects measured home BP before taking antihypertensive medication. The antihypertensive treatment regime (frequency and timing) was not changed during the study period. 4 patients were excluded. The inclusion criteria were aged between 18 and 75 years and the presence of written consent to participate in the study. The exclusion criteria were significant arrhythmias, pregnancy and lactation. The University of Bonn issued a positive ethical vote on the study.

### Methods of blood pressure measurement

#### 1. Office blood pressure measurement (office BP)

The blood pressure was measured in the doctor's office in accordance with the recommendations of the American Heart Association (AHA) and the European Society of Hypertension (ESH) after the participant had rested for five minutes in a sitting position [6,8]. On study day 1, two measurements per upper arm were made with a two-minute interval between each measurements with the auscultatory blood pressure instrument from Acuson (Acuson Greenlight 300 with universal cuff). Office BP was measured with the same procedure only on the left upper arm on study day 7. The office BP was taken with a precision nearest 2 mmHg.

#### 2. Blood pressure self-measurement (home BP)

The patients measured their blood pressure themselves with the oscillometric wrist instrument from Omron (wrist instrument HEM- 637 IT with position sensor). Between study days 1 and 7 and study days 8 and 14, the participants took two measurements on the left wrist each day in the morning between 6 and 9 a.m. and in the evening between 8 and 9 p.m. after sitting for five minutes with a two-minute interval between the two consecutive measurements. Hypertensive subjects measured home BP before taking antihypertensive medication. The antihypertensive treatment regime was not changed during the study period. Patients were instructed how to correctly use the oscillometric Omron wrist device for home BP measurement with a standardized 30 min. teaching program. The blood pressure parameters of the first week (day 1 to day 7) were compared with the results of the second week (day 8 to day 14). The HEM- 637 IT wrist device is validated according to the international protocol of the European Society of Hypertension [9]. The home BP was measured in accordance with the user procedures and recommendations of the European Society of Hypertension [8].

#### 3. Ambulatory 24-hour-blood pressure measurement (24-h ABPM)

The ambulatory 24-h blood pressure was performed according to AHA and ESH guidelines and measured with the ABPM 04 instrument of the company Meditech, Hungary, on the left upper arm on study day 7 and 14. In the period between 6 a.m. and 10 p.m., an oscillometric blood pressure measurement was taken automatically at intervals of 20 minutes, and at intervals of 45 minutes in the period between 10 p.m. and 6 a.m. The CardioTens is validated according to the BHS (British Hypertension Society) protocol [10]

### Echocardiography

On study day 1, a cardiac ultrasonographic investigation was conducted in all patients with the Hewlett Packard instrument (HP 5500, USA) with a 2.5 MHz transducer. The left ventricular muscle mass index (LVMI) in g/m^2 ^body surface was calculated in accordance with the corrected Devereaux formula. LVMI was analysed by an investigator blinded to the results of the BP measurements.

### Statistics

The parameters investigated were anthroprometric data such as age, gender, height, body weight, body mass index, circumference of the wrist and of the upper arm. The parameters of office BP were systolic and diastolic blood pressure and pulse pressure. The parameters of home BP were systolic and diastolic blood pressure, heart rate as well as pulse pressure. The parameters of the ambulatory 24-h ABPM were systolic and diastolic blood pressure, heart rate, and pulse pressure measured, for the total ambulatory 24 hour period, for the daytime (6 a.m. to 10 p.m.) and for the nighttime (10 p.m. to 6 a.m.).

Before the evaluation, the systolic and diastolic blood pressure values were averaged for each method of measurement (measurement in the doctor's office, 24-h measurement and self-measurement) over the respective visit to the doctor's office, day of measurement or the respective week of measurement. Consequently, two values (mean values) were available for each of the methods. These had been measured at an interval of about one week. The mean values determined in this way were the basis for the descriptive evaluation of the values measured with the different methods.

In order to be able to compare the reproducibility of the three measurement strategies, the standard deviation of mean differences were compared. Student's t-test was used to statistically compare the SD of mean difference between measurement methods. Furthermore, the squared differences between the first and second set of BP measurements was calculated for office, home and 24-h ABPM.

The left ventricular mass was used as a measure for the extent of end organ damage and the correlation coefficient of the blood pressure parameters obtained with various methods of measurement with the left ventricular mass index was calculated.

For each BP measurement method, the number of patients needed to detect a two-sided α risk of 5% and a statistical power of 80% to detect a systolic and diastolic blood pressure difference of 5 mm Hg was calculated.

## Results

The data from 97 patients were evaluated to compare the reproducibility of blood pressure measurements. The correlation analysis of blood pressure parameters with the left ventricular mass index could only be carried out with a subgroup of 74 patients in whom satisfactory sonography was possible. The patients' characteristics are shown in Table 1.

**Table 1:** Patient characteristics

	Range	Mean	SD
Age (years)	19–74	52	12
Height (cm)	153–199	173	9
Weight (kg)	52–128	81	17
BMI(kg/m^2^)	19–43	27	5
Left ventricular mass index (g/m^2^)	53–189	101	27
Left wrist circumference (cm)	13.5–20	17	2
Left upper arm circumference (cm)	23–41	30	3

SD: Standard deviation

Compared to study days 1 and 7, there was no difference in the office BP values. There was also no difference in the home BP values, compared to week 1 and week 2, and no diference in the 24-h ABPM values compared to study 7 and 14. The blood pressure values obtained with the different methods are shown in table 2.

**Table 2:** Blood pressure values obtained with the different methods for the two measurement periods

Blood pressure (mmHg)	Mean systolic	SD	Mean diastolic	SD
Office BP Day 1	148	24	92	13
Office BP Day 7	144	21	90	12
Home BP Week 1	135	16	84	12
Home BP Week 2	134	16	83	12
24-h ABPM Day 7	131	15	79	11
24-h ABPM Day 14	133	16	80	11
24-h ABPM Daytime day 7	136	16	83	11
24-h ABPM Daytime day 14	137	17	84	12

SD: Standard deviation

The standard deviation of mean differences of the respective measurement method is shown in Table 3. This is a measure for the reproducibility of the respective method of measuring blood pressure.

**Table 3:** Standard deviation of the mean differences between the two measurement periods

	systolic		diastolic	
	
	SD	95% CI	SD	95% CI
Home BP	3.81	3.34 – 4.44	2.77	2.43 – 3.22
24-h ABPM	7.83	6.90 – 9.17	4.09	3.59 – 4.77
Office BP	8.10	7.10 – 9.43	4.76	4.18 – 5.54
p- value				
Home vers. 24-h ABPM	p < 0.001		p = 0.127	
Home vers. Office BP	p < 0.001		p < 0.001	
Office vers 24-h ABPM	p = 0.80		p = 0.10	

SD: Standard deviation of mean differences. 95% CI: 95% confidence interval.

For the three methods of blood pressure measurement, the standard deviation of mean differences reveals that home BP shows the least variability both for systolic and diastolic BP. The reproducibility of systolic and diastolic home BP was significantly higher than the reproducibility of office BP (p = 0.001 systolic BP, and p < 0.001 diastolic BP) and the reproducibility of 24-h ABPM (p < 0.001 systolic BP, p = 0.127 diastolic BP). The reproducibility of systolic and diastolic office and 24-h ABPM was not significantly different (p = 0.8 systolic, p = 0.1 diastolic).

**Figure 1:**
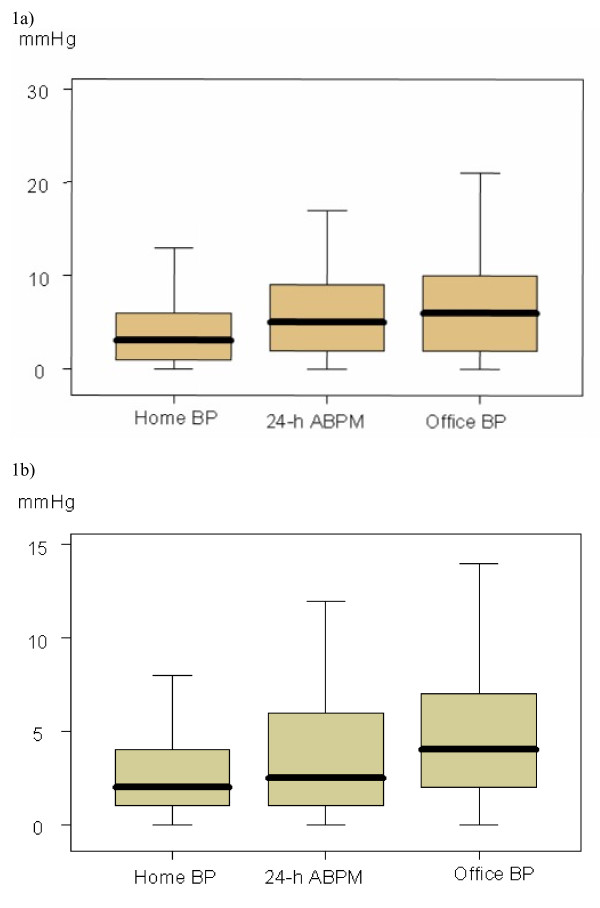
**Reproducibility of measurements shown as squared differences (y-axis) between the first and second set of blood pressure (BP)measurements for Home BP, 24-h- ABPM and Office BP**. Values are shown as box plots with median, 25/75 percentiles and minimum/maximum values. 1a: Squared systolic BP differences 1b.: Squared diastolic BP differences.

The squared differences for office, home BP and the 24-h ABPM was analyzed as a further parameter for appraising the reproducibility of the different methods of BP measurement. The results are shown in figure 1a and 1b. Both the variation for the systolic and the diastolic BP is lowest for home BP measurement.

The results for reproducibility did not change, if a separate analysis was performed for treated or untreated patients.

**Figure 2:**
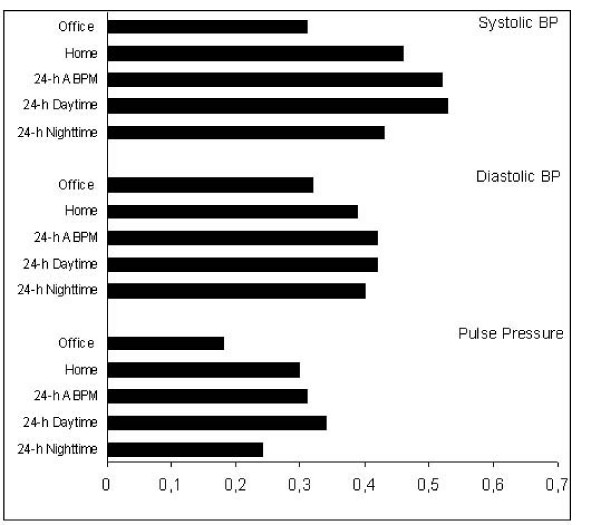
**Correlation coefficient of the blood pressure parameters with the left ventricular mass index (LVMI)**. The correlation coefficient of 24-h ABMP systolic BP (r = 0.52) with left ventricular mass index was significantly higher than with systolic office BP (r = 0.31) (p = 0.035). The difference between 24-h ABPM and home BP (r = 0.46) was not significant (p = 0.456). The correlation coefficient with LVMI was not significantly different between the tree measurement methods for diastolic BP.

The correlation coefficient of the BP parameters of different methods of measuring BP with the left ventricular mass index is shown in figure 2. The correlation coefficient of 24-h ABMP systolic BP (r = 0.52) with left ventricular mass index was significantly higher than with systolic office BP (r = 0.31) (p = 0.035). The difference between 24-h ABPM and home BP (r = 0.46) was not significant (p = 0.456). The correlation coefficient with LVMI was not significantly different between the three measurement methods for diastolic BP (fig. 2)

For each blood pressure measurement method the number of patients needed to detect a two-sided α risk of 5% and a statistical power of 80% a systolic and diastolic blood pressure difference of 5 mm Hg is shown in table 4.

**Table 4:** Sensitivity of BP methods to measure significant BP changes

	Number of Patients	
Blood pressure method	Systolic	Diastolic

home BP	17	10
24-h ABPM	65	19
Office BP	64	23

Number of patients needed to detect a two-sided α risk of 5% and a statistical power of 80% a systolic and diastolic blood pressure difference of 5 mm Hg.

## Discussion

In the present study, it could be shown that home BP measurements at the wrist using a validated instrument with activated position sensor show very much better reproducibility not only compared to conventional office BP measurements, but also compared to standardized 24-h ABPM. Moreover, the coefficients of correlation with left ventricular mass index as an indirect parameter of hypertensive end organ damage were comparable with those of home BP recently carried out on the upper arm and did not differ significantly from those of 24-h ABPM.

As shown by earlier studies, the reproducibility of BP measurements can be substantially increased by 24-h ABPM compared to office BP [1,2,11]. However, the reproducibility of 24-h ABPM was significantly superior to home BP in previous studies [2]. A later investigation with clinically validated home instruments and standardized measurement protocols showed a distinctly better reproducibility with home BP compared to office BP [4]. This is taken to be essentially an effect of the larger number of measurement values owing in home BP, an average of approximately 28 self-measurements being made per week depending on the measurement protocol. For example the standard deviations of the mean difference between two measurement periods can be used as an expression of individual measurement variability. The standard deviation of the mean difference both in 24-h ABPM and in home BP essentially depends on the number of measurements [12]. As has been shown by previous studies, this standard deviation of the mean differences for office BP is between 10 and 17 mmHg for systolic and 7–10 mmHg for diastolic values. This deviation is reduced to 7–9 mmHg systolic and 5–7 mmHg diastolic by 24-h ABPM. The scale of multiple home BP measurement and their effect on this standard deviation is similar to that in 24-h ABPM. Our working group found values of 5.4 mmHg for systolic and 4.3 mmHg for diastolic standard deviations of the mean differences [4]. In a pharmacological study in 1992, we were also able to show that the reproducibility of upper-arm measurements at home was better than that of office BP measurement, but also better than standardized 24-h ABPM [13]. In this study, the sample size was very much reduced both compared to office BP and also compared to 24-h ABPM [13]. In consequence of this study as well as further studies, standardized home BP have been accorded an ever greater significance in clinical and pharmacological studies [14-16].

Also in the present study, for each BP measurement method, we have calculated the number of patients needed to detect a two-sided α risk of 5% and a statistical power of 80% to detect a systolic and diastolic blood pressure difference of 5 mm Hg (table 4). The results of our study indicate, that home blood pressure measurement with a validated wrist device and activated position sensor can improve the sensitivity of blood pressure measurement in clinical trials by reducing the number of patients needed to detect clinically significant differences in blood pressure changes.

The present study was carried out with a validated wrist blood pressure instrument with an activated position sensor [17-19]. According to a recently published study, the measurement precision of this system is further improved by activation of the wrist position sensor [20]. However, current recommendations from national and international societies do not recommend wrist measurement as the method of choice for performance of home BP [21,22]. This is mainly due to the fact that numerous imprecise and unvalidated wrist instruments were on the market in the past. Moreover, the measurement precision of wrist measurement is altered by the position of the wrist in relation to that of the heart. Newer technologies offer innovative approaches to improving the position of measurement by use of an activated position sensor [21,23]. Moreover, the system we used allows automatic storage of all self-measured values, avoiding the familiar errors of transfer in digital display or print-outs [24].

The standard deviations for the mean differences are between 5 and 8 mmHg systolic and 4 and 6 mmHg diastolic for home BP with upper-arm instruments. A recently published study of Stergiou demonstrated that the reproducibility was indeed even better with a newer validated upper-arm instrument with digital print-outs and standardized measurement than with a standardized 24-h ABPM [5]. The SD systolic and diastolic blood pressures between the two sets of measurements also showed that in this study home BP with a wrist instrument in terms of reproducibility appeared to be superior to office BP as well as 24-h ABPM.

The absolute number of values measured does not provide a cogent explanation for the better reproducibility of home BP, since both in the first and in the second week an average of 31 ± 7 and 31 ± 9 measurement values were obtained of home BP as compared to 52+8 and 51 ± 8 measurement values in 24-h ABPM. It is more likely that the largely standardized wrist measurement with automatic recording of the values measured, activated position sensor and measurements and defined times of day have contributed to the very good reproducibility. Moreover, fluctuations in BP which are not seen in 24-h ABPM but might contribute to a raised variability are also detected over a week with home BP. In our investigation, we have also deliberately included the first day of home BP in the analysis, since the patients were already familiar with the instrument as a result of prior thorough instruction in self-measurement at the wrist. However, the inclusion of the first day in the measurement analysis did not affect the reproducibility owing to the large number of measurements.

Besides the reproducibility, the correlation of the different methods of BP measurement with hypertensive end organ damage measured with the left ventricular mass index was also investigated in the present study. According to the study results available so far, the 24-h ABPM shows correlation coefficients with the left ventricular mass index of 0.52 for systolic and 0.46 for diastolic values [3,25,26]. Similar to the reproducibility, this better correlation with end organ damage compared to the office BP is essentially influenced by the greater number of measurements carried out during 24-h ABPM. The day-night difference which is detected with the 24-h ABPM does not contribute substantially to the variability of the left ventricular mass index [3]. Recently published studies have demonstrated that multiple standardized measurements carried out by a trained nurse in the hospital reveal similar coefficients of correlation with the left ventricular mass index or the intima media thickness of the carotid artery which are similar to that of 24-h ABPM [27,28]. Earlier studies with home BP have already shown a better correlation with hypertensive end organ damage compared to office BP [29-31]. The coefficients of correlation between home BP and LVMI on the one hand and 24-h ABPM and LVMI on the other hand calculated in the present study did not reveal any significant differences. However there was slight but not significant trent towards higher correlations of LVMI with 24-h ABPM in the present study. The correlation coefficients with LVMI determined with the wrist instrument corresponded to those in recently published upper-arm studies.

## Conclusion

In this study, we attempted to include as large as possible a spectrum of BP values in the range between optimal BP values and hypertension of severity grade III. For this purpose, we considered it necessary also to include in the investigation patients under antihypertensive treatment who were referred to our hospital because of hypertension that was refractory to treatment. However, since the antihypertensive medication remained unchanged over the investigation period in all patients, an effect on one of the methods of BP measurement would not have been expected.

The present study did not address the question whether home BP measurement with a wrist device with position sensor is more or less reproducibile compared to an upper arm device. In our view, the study design for such a study would be very difficult to handle. Therefore, this question may be evaluated in future studies

Furthermore, it was not our purpose to advocate the general use of wrist devices. Such devices have physiological limits with regards to accuracy and only devices with a position sensor may be considered for clinical use [8]. Even though this wrist device was reproducible in the research setting, it may be inaccurate if the instructions are not strictly followed in the usual setting.

To summarize, the present study showed that the wrist measurement of home BP with a validated system comprising an activated position sensor was superior in respect of reproducibility both to office BP and 24-h ABPM. Moreover, a correlation of the wrist measurement with hypertensive end organ damage similar to that with 24-h ABPM was revealed. Although wrist devices have to be used cautious and with defined limitations, the use of validated devices with position sensor according to recently recommended measurement schedules [9] might have the potential to be used for therapy monitoring in patients who prefer this method.

## Competing interests

This study was supported by Omron Company, Japan.

The article-processing charge will be financed by Omron Company

All authors declare that they have no competing interests

## Authors' contributions

SU conceived of the study and its coordination, performed the echocardiography, RF performed the statistical analysis. MB recruited the patients, supplied blood pressure measurements. GN drafted the manuscript, TM evaluated the echocardiography and drafted the manuscript. All authors read and approved the final manuscript.

## Pre-publication history

The pre-publication history for this paper can be accessed here:


